# Microbiological Effect of Essential Oils in Combination with Subgingival Ultrasonic Instrumentation and Mouth Rinsing in Chronic Periodontitis Patients

**DOI:** 10.1155/2013/146479

**Published:** 2013-09-19

**Authors:** Toshiya Morozumi, Takehiko Kubota, Daisuke Abe, Taro Shimizu, Kaname Nohno, Hiromasa Yoshie

**Affiliations:** ^1^Division of Periodontology, Department of Oral Biological Science, Niigata University Graduate School of Medical and Dental Sciences, 2-5274 Gakkocho-Dori, Chuo-ku, Niigata 951-8514, Japan; ^2^Division of Preventive Dentistry, Department of Oral Health Science, Niigata University Graduate School of Medical and Dental Sciences, 2-5274 Gakkocho-Dori, Chuo-ku, Niigata 951-854, Japan

## Abstract

Thirty chronic periodontitis patients were randomly assigned to 3 groups: control, saline, and essential oil-containing antiseptic (EO). Subgingival plaque was collected from a total of 90 pockets across all subjects. Subsequently, subgingival ultrasonic instrumentation (SUI) was performed by using EO or saline as the irrigation agent. After continuous mouth rinsing at home with EO or saline for 7 days, subgingival plaques were sampled again. Periodontopathic bacteria were quantified using the modified Invader PLUS assay. The total bacterial count in shallow pockets (probing pocket depth (PPD) = 4-5 mm) was significantly reduced in both saline (*P* < 0.05) and EO groups (*P* < 0.01). The total bacterial count (*P* < 0.05) and *Porphyromonas gingivalis* (*P* < 0.01) and *Tannerella forsythia* (*P* < 0.05) count in deep pockets (PPD ≥6 mm) were significantly reduced only in the EO group. In comparisons of the change ratio relative to baseline value of total bacteria counts across categories, both the saline and EO groups for PPD 4-5 mm and the EO group for PPD 6 mm showed a significantly low ratio (*P* < 0.05). The adjunctive use of EO may be effective in reducing subgingival bacterial counts in both shallow and deep pockets. This trial is registered with UMIN Clinical Trials Registry UMIN000007484.

## 1. Introduction

It is well established that periodontitis is caused by local bacterial infection from pathogenic microorganisms that colonize and proliferate in the gingival crevice and periodontal pockets of a susceptible host [1, 2]. Periodontal therapy is aimed at eliminating the dental biofilm and calculus by means of mechanical removal [3]. However, the efficacy of mechanical therapy is reduced with an increasing pocket depth and furcation involvement; the deeper the pocket and the greater the furcation involvement, the more deposits are left behind [4]. Thus, the use of an antiseptic in conjunction with mechanical instrumentation has been widely applied to control the subgingival biofilm. Many clinical and microbiological studies have been conducted; however, their findings are inconclusive [5, 6]. 

Various antiseptics, including chlorhexidine and povidone-iodine, have been hitherto used as irrigating agents. Although chlorhexidine is considered the most efficient antimicrobial agent for supragingival plaque control, its use as an irrigant in combination with subgingival ultrasonic instrumentation (SUI) is not associated with additional clinical and microbiological benefits [7–9]. Povidone-iodine has a beneficial effect as a solution for subgingival irrigation [10, 11]; however, the antiseptic is associated with an increased risk of hypersensitivity reaction, and its prolonged use is associated with thyroid dysfunction and staining of the tooth surface and mucosa [12–14].

Essential oil-containing antiseptic (EO) is an over-the-counter mouth wash containing 2 phenol-related essential oils; EO is associated with only minimal side effects and kills a wide range of microorganisms by disrupting their cell walls and inhibiting their enzyme activity [15, 16]. EO is capable of extracting bacterial endotoxins, which theoretically may reduce plaque pathogenicity [17]. Moreover, *in vitro* and *in vivo* studies have shown that EO penetrates the plaque biofilm and is active against biofilm-embedded bacteria [15, 18]. These characteristics may support the potentiality of EO as a subgingival irrigating agent. 

Although there is accumulating evidence on the efficacy and safety of EO when used for gingivitis and supragingival plaque control [19–21], insufficient information is available regarding its effect on subgingival microbiota. In addition, in those studies, EO was either applied by using home subgingival irrigation devices by the patients themselves or by professional irrigation with syringes. Few studies have evaluated the effects of SUI irrigated with EO on microbiota. Recently, we have reported that a combination of SUI and 7-days mouth wash using EO significantly reduced subgingival bacterial counts of total bacteria, *Porphyromonas gingivalis* (*P. gingivalis*), and *Tannerella forsythia* (*T. forsythia*) [22]. However, it is still unclear about the difference of the efficacy by the depth of the pocket. This question has not been adequately discussed and is worth investigating. In the present study, we therefore evaluated the microbiological effects of EO combined with SUI and mouth rinsing in various degrees of periodontal pocket depths in chronic periodontitis patients.

## 2. Materials and Methods

### 2.1. Subjects

Thirty subjects were recruited from patients attending the Niigata University Medical and Dental Hospital, Niigata, Japan, between January 2007 and October 2008. The study protocol was approved by the regional ethical committee of the Faculty of Dentistry, Niigata University (number 110, on December 11, 2006), and all subjects provided informed consent before participating in the study. Systemically healthy subjects with a minimum of 20 teeth and generalised moderate-to-severe chronic periodontitis, which was defined as having at least 3 teeth with a probing pocket depth (PPD) of ≥4 mm in each quadrant, were selected. 

Subjects with the following conditions were excluded: patients who had taken systemic antibiotics, anti-inflammatory drugs, or immunosuppressive drugs within 3 months before the experiment. Subjects who had received periodontal treatment within 6 months before the experiment, those who were regularly using an oral irrigation device and/or mouth rinse, and those who had incompatible dentition (e.g., orthodontic bands, partial dentures, or teeth unsuitable for extensive ultrasonic scaling) were also excluded from the study. 

### 2.2. Clinical Examination

A thorough medical and drug history was obtained for each patient. Smoking habits were also recorded (number of cigarettes/day, years of smoking). For more than 1 month before the study, all subjects received standard oral hygiene instructions and underwent full-mouth supragingival scaling and eventually showed a plaque control record of <20%. One week before the start of the study, a full-mouth periodontal examination was performed. The following clinical parameters were recorded: PPD, clinical attachment level (CAL), and bleeding on probing. PPD and CAL were recorded at 6 sites per tooth (mesiobuccal, buccal, distobuccal, mesiolingual, lingual, and distolingual) with a periodontal probe (CP-12 Color-Coded Probe; Hu-Friedy, Chicago, IL, USA). The quadrant exhibiting the most severe periodontal condition on the basis of clinical findings was selected as the site for SUI.

### 2.3. Clinical Protocol

Sample size determination was performed with reference to our previous report before the study was initiated [22]. A total of 30 subjects were randomly assigned to 3 groups based on the treatment protocol (control, *N* = 10; saline, *N* = 10; EO, *N* = 10) by using random tables provided by one of the authors (D.A.) and were given a code number for identification throughout the study. All experimental procedures and collection of clinical data were performed in the dental clinic in Niigata University Medical and Dental Hospital between February 2007 and December 2008.

After the selection of the 3 deepest pockets in the treated quadrant, subgingival plaque was collected at baseline from a total of 90 periodontal pockets across all 3 groups. Subsequently, quadrant SUI was performed using an ultrasonic device (Suprason P-Max with an irrigation kit; Satelec, Bordeaux, France) and specific tips (HY1 tip; Satelec), which involved irrigation with 100 mL EO (EO group; Fresh Mint Listerine; Johnson and Johnson K. K. Consumer Company, Tokyo, Japan) or sterile saline (saline group; Otsuka normal saline; Otsuka Pharmaceutical Co., Ltd., Tokyo, Japan) for 10 min. Following SUI, subjects were asked to perform mouth rinsing at home 4 times daily (after each meal and at bedtime) for 20 s with 20 mL EO (EO group) or saline (saline group) for 7 days according to our previous report, which was a modified dosage of the manufacture's recommendation [22]. No treatment was performed in control group subjects. After 7 days, subgingival plaque was collected again in all 3 groups. All clinical procedures including periodontal examination and SUI were performed by a single dentist (T. M.) who was sufficiently trained to prevent technical inconsistencies. Subjects were requested not to brush their teeth and to consume only liquids for at least 2 h before sampling.

### 2.4. Subgingival Plaque Sampling and Quantitative Bacterial Assay

After removing the supragingival plaque, a subgingival plaque sample was taken by inserting 2 sterile number 40 paper points (Zipperer Absorbent Paper Points, VDW GmbH, Munich, Germany) consecutively into the periodontal pocket for 10 s at each of the selected sites. 

Quantitative analysis of the total bacterial count and periodontopathic bacterial count, including *P. gingivalis*, *Prevotella intermedia* (*P. intermedia*), and *T. forsythia*, was performed using a modification of the Invader PLUS assay [22–24]. Briefly, bacterial DNA was extracted from the plaque samples suspended into 1 mL of PBS using the MagNA Pure LC Total Nucleic Acid Isolation Kit (Roche, Basel, Switzerland). The individual sequences of each bacterial species were obtained from a public database (National Center for Biotechnology Information, Bethesda, MD, USA). Primers for each species were designed based on a region of the 16S rRNA gene. A pair of universal primers and a universal probe was used for the total number of bacteria. Primary probes and Invader oligos were designed using Invader technology creator (HOLOGIC, Madison, WI, USA) and were based on sequences in the amplified regions [25]. 

Template DNA was added to a 15 *μ*L reaction mixture containing primers for each species, 50 *μ*M dNTP, 700 nM primary probe, 70 nM Invader oligo, 2.5 U polymerase chain reaction (PCR) enzyme (EagleTaq DNA polymerase, Roche), and the Invader core reagent kit (Cleavase XI Invader core reagent kit, HOLOGIC) containing a fluorescence resonance energy transfer (FRET) mix and an enzyme/Mgcl_2_ solution. The reaction mixture was preheated at 95°C for 20 min, and a 2-step PCR reaction was performed for 35 cycles (95°C for 1 s and 63°C for 1 min) using an ABI PRISM 7900 thermocycler (Applied Biosystems, Foster City, CA, USA). Fluorescence values of carboxyfluorescein (FAM; wavelength/bandwidth: excitation, 485/20 nm; emission, 530/25 nm) were measured at the end of the incubation/extension step at 63°C for each cycle. 

The limit of detection for this method was determined for each species with dilutions of bacterial DNA. Standard curves were constructed based on a crossing point determined by the fit point method.

### 2.5. Statistical Analyses

Descriptive analysis was conducted (mean and standard deviation (SD)) for the collected data. Differences in clinical parameters between the groups were established using the Kruskal-Wallis test. Intragroup comparisons of subgingival organisms were performed using the Wilcoxon signed-rank test. Intergroup comparisons of subgingival organisms were conducted using analysis of variance (ANOVA) and Bonferroni/Dunn's test as post hoc test. Results were analysed by SPSS version 12.0 statistical software for Windows (SPSS Inc., Chicago, IL, USA). A *P* value < 0.05 was considered statistically significant.

## 3. Results

### 3.1. Subject Description

All participants successfully completed the study protocol. Any subject was not lost due to PPD reduction after the 1 month plaque control. None of the subjects reported any general health problems throughout the study. The clinical and demographic characteristics of the study subjects are shown in Table 1; no statistically significant differences were observed between the groups. Meanwhile, statistically significant differences were seen in all clinical characteristics of the sampled sites between shallow and deep pockets (Table 2).

### 3.2. Bacteria in Subgingival Plaque

Means ± SD subgingival bacterial counts in shallow (PPD = 4-5 mm) and deep (PPD ≥ 6 mm) pockets at baseline and after 7 days are shown in Tables 3 and 4. PPD categories are based on those proposed by Rosling et al. [11].

For shallow pockets, the bacterial count of all species observed after 7 days in the control group was similar to baseline values. Only the total bacterial count in the saline group was significantly decreased (*P* < 0.05). The EO group showed significant reductions in total bacterial count and *P. gingivalis* and *T. forsythia* count (*P* < 0.01). 

For deep pockets, in contrast to the significant increase in total bacterial count and *P. gingivalis *count observed in the control group (*P* < 0.05), significant decrease in total bacterial count (*P* < 0.05) and *P. gingivalis* (*P* < 0.01) and *T. forsythia* (*P* < 0.05) count was seen in the EO group. There were no significant differences for any of the species in the saline group.

**Figure 1:**
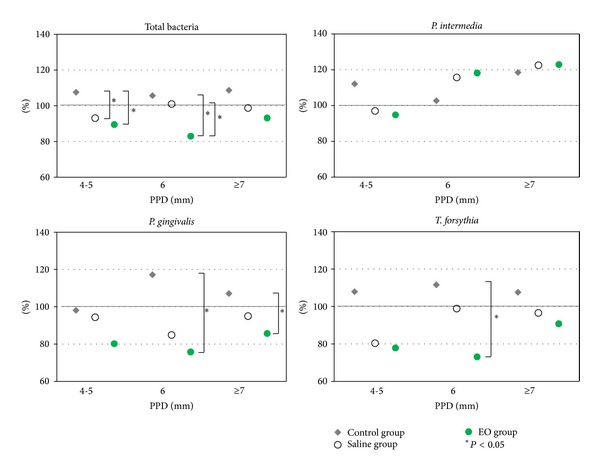
Intergroup comparisons of the change ratio of subgingival bacterial counts. Intergroup comparisons of percentage changes ratio of subgingival bacterial counts after 7 days relative to the baseline value for total bacteria and selected periodontitis-related bacteria across the 3 categories of periodontal pocket depth. * indicates statistical significance at *P* < 0.05.

Figure 1 shows the results of intergroup comparisons of the change ratio of subgingival bacterial counts after 7 days relative to the baseline value for total bacteria and selected periodontal bacteria when periodontal pockets were classified into 3 PPD categories (4-5 mm, 6 mm, ≥7 mm). Both the saline and EO groups had a significantly lower total bacterial ratio than the control group for a PPD of 4-5 mm (*P* < 0.05); however, there was no significant difference for each individual species. For a PPD of 6 mm, only the EO group showed a significantly lower total bacterial ratio and *P. gingivalis* and *T. forsythia* ratio than the control group (*P* < 0.05). In particular, the total bacterial ratio in the EO group was significantly lower than that in the saline group (*P* < 0.05). For a PPD of ≥7 mm, the *P. gingivalis* ratio was significantly lower in the EO group than in the control group (*P* < 0.05); however, there was no significant difference in total bacterial ratio and that of the other species. The *P. intermedia* ratio for all PPD categories was comparable between the 3 groups. 

## 4. Discussion

The objective of the present study was to assess the microbiological effect of EO in combination with SUI and mouth rinsing for various periodontal pocket depths. We demonstrated that a combined treatment with SUI and mouth rinsing with EO was effective in reducing subgingival bacterial counts in both shallow and deep pockets. 

It has been reported that smoking may detrimentally affect the composition of subgingival microflora [26]. However, in the present study, no significant difference was observed between smokers and nonsmokers with respect to subgingival bacterial counts. This may be because of the inclusion of light smokers (mean 9.4 pack years) and the use of sampling sites that are minimally affected by smoking. 

In this study, the total bacterial count in shallow pockets (PPD = 4-5 mm) was significantly reduced in both the saline and EO group. However, the reduction in the EO group was drastic, with a significant reduction of periodontopathic bacteria such as *P. gingivalis* and *T. forsythia* seen only in the EO group. In contrast, it was observed that only the EO group showed a significant reduction in microbial counts when deep pockets (PPD ≥ 6 mm) were analysed. The change ratio of total bacterial count in EO group was also significantly lower than that in the other groups at PPD of 4-5 and 6 mm. These results suggest that a combination use of SUI and 7-day mouth wash with EO was effective in reducing subgingival bacterial amount in both shallow and deep pockets. In addition, Feng et al. recently have reported that a significantly greater CAL gain and PPD reduction was observed by SUI irrigated with EO compared to that of negative solutions in deep pockets (PPD ≥ 7 mm) [27]. This data imply the clinical efficacy of EO in deep periodontal pockets. 

The experimental protocol was referred to in our previous report, which demonstrated the decreased bacterial counts in the periodontal pockets due to a combination of SUI and 7-day mouth wash using EO. In the present study, we have further evaluated the subgingival microbiota among 3 groups-control, EO, and a newly introduced saline group-in order to assess whether mechanical or chemical effects were dominant. The saline group showed a significant reduction of total bacterial counts in shallow pockets. This indicates that a combination of SUI and mouth rinsing without an antiseptic also has some effect on reducing subgingival microorganisms in shallow pockets. It is probable due to subgingival plaque removal and bacterial cell disruption achieved by the vibrating chipping action of the tip, cavitational activity, and acoustic microstreaming of SUI. In the EO group, it is assumed that these mechanisms improve the bactericidal effect of EO, leading to a greater reduction in subgingival microflora [28, 29]. Further, more frequent mouth wash in this protocol could partly influence the reductions. 

Interestingly, intragroup and intergroup comparisons showed no significant difference with respect to *P. intermedia* counts. This may be attributable to the low bacterial counts of approximately 1/100–1/10 as compared to those of other bacteria examined in this study. These data are consistent with the findings of Rhemrev et al. [30] and Botero et al. [31], who reported that *P. intermedia* counts in the sulcus of diseased chronic periodontitis sites are very low even though the detection rate is high. The likelihood that *P. intermedia *is resistant to EO seems to be remote.

Bacterial counts in the EO group significantly decreased after 7 days in both shallow and deep pockets. In the short term study, however, SUI could profoundly affect the subgingival microbiota. Fine et al. reported the effect of the continual use of oral irrigation device with EO at home once daily for 6 weeks [20]. Bacterial counts of periodontal bacteria species including *Fusobacterium *sp., *Capnocytophaga *sp., *Streptococcus sanguinis,* and *P. intermedia* were significantly decreased compared to the baseline after 1 or 3 weeks; however, it was comparable after 3 or 6 weeks. Thus, it seems to be difficult to keep the subgingival bacteria low for long term because of their regrowth.

In the study design, the biofilm on the supra- and subgingival plaque was first expected to be destroyed by mechanical treatment based on SUI, resulting in a reduction in the total number of bacteria. Then, it is anticipated that daily mouth washing with EO after SUI disrupts the contiguous supragingival plaque and the regrowth of subgingival bacteria would be kept to a minimum. Studies have demonstrated that mouth rinsing with EO can effect changes in the subgingival flora within periodontal pockets [32, 33]. While, according to a report by Boyd et al., a mouth wash using a dental plaque-disclosing solution could penetrate only as deep as 0.1 mm into the periodontal pockets [34], this may suggest that the involvement of mouth rinsing for subgingival microbiota counts is lightly affected. Therefore, we speculate that mouth rinsing with EO possesses a certain effect on the subgingival flora although the degree is limited.

## 5. Conclusions

The results of the present study suggest that the adjunctive use of EO to a combination of SUI and mouth rinsing is effective in reducing subgingival bacterial counts in both shallow and deep pockets. This study is the first reporting the effective range of periodontal pocket depths by a combined treatment with SUI and mouth rinsing using EO. As further studies, correlation between bacterial and clinical data in some time points, change of oral hygiene level based on plaque index score, and several comparisons in further classified groups will provide beneficial information for this combination therapy. Studies across different categories of periodontal pocket depths are also necessary to verify the potential efficacy of this adjunctive therapy. In addition, the development of novel antibacterial substances such as enzymes and peptides, which can interfere with bacterial activity, is an issue for future research. 

## Figures and Tables

**Table 1:** Demographic and clinical characteristics of the study population (mean ± SD).

Variable	Control group (*N* = 10)	Saline group (*N* = 10)	EO group (*N* = 10)
Age (years)	55.4 ± 9.3	55.6 ± 10.0	54.1 ± 8.7
Gender (*n*)			
Male/female	8/2	6/4	6/4
Smoking (*n*)			
Smoker/nonsmoker	4/6	3/7	3/7
Number of teeth	24.0 ± 3.2	24.2 ± 3.9	23.4 ± 2.5
PPD (mm)	15/10/5	16/9/5	15/8/7
CAL (mm)	3.7 ± 0.7	3.8 ± 0.8	4.0 ± 0.9
BOP (% positive)	3.8 ± 0.5	3.9 ± 0.7	4.0 ± 1.0
	52.0 ± 18.7	54.3 ± 16.6	52.9 ± 15.9

EO: essential oil-containing antiseptic; PPD: probing pocket depth; CAL: clinical attachment level; BOP: bleeding on probing. No statistically significant difference at the 0.05 level was observed for any variables between the groups (Kruskal-Wallis test).

**Table 2:** Clinical characteristics of sampling sites.

Variable	Shallow pockets (PPD = 4-5 mm)			Deep pockets (PPD ≥ 6 mm)		
	Control group	Saline group	EO group	Control group	Saline group	EO group
Number of sites (*n*)	8	12	12	22	18	18
Type of the teeth						
Anterior/premolar/molar	3/4/1	5/4/3	6/4/2	12/6/4	10/5/3	8/8/2
Furcation involvement (*n*)	0	0	0	2	1	1
PPD (mm)	5.0 ± 0.0	4.9 ± 0.3	5.0 ± 0.4	7.0 ± 1.2	6.7 ± 1.7	7.2 ± 1.6
CAL (mm)	5.4 ± 0.7	5.5 ± 0.7	5.3 ± 0.7	7.8 ± 1.5	7.6 ± 1.8	7.9 ± 1.7
BOP (% positive)	75.0	83.3	75.0	100	100	100

EO: essential oil-containing antiseptic; furcation involvement (*n*), number of teeth with furcation involvement; PPD: probing pocket depth; CAL: clinical attachment level; BOP: bleeding on probing. Values are expressed as mean ± SD. No statistically significant difference was observed for any variables between the 3 groups (Kruskal-Wallis test). Statistically significant difference was observed for all variables between shallow pockets and deep pockets (*P* < 0.05, Mann-Whitney *U* test).

**Table 3:** Comparison of subgingival bacterial counts in shallow pockets (PPD = 4-5 mm).

		Baseline	After 7 days	*P* value
Control group *n* = 8 sites	Total bacteria	5.2 ± 0.7	5.6 ± 0.8	0.1604
	*P. gingivalis *	3.3 ± 1.9	3.2 ± 2.2	0.5992
	*P. intermedia *	1.9 ± 1.2	2.3 ± 1.6	0.2249
	*T. forsythia *	3.8 ± 1.0	4.0 ± 1.5	0.2603

Saline group *n* = 12 sites	Total bacteria	5.5 ± 0.9	5.1 ± 1.0	0.0356*
	*P. gingivalis *	3.3 ± 1.8	2.8 ± 1.7	0.1139
	*P. intermedia *	2.6 ± 1.2	2.4 ± 1.4	0.7210
	*T. forsythia *	3.6 ± 1.0	2.9 ± 1.5	0.0711

EO group *n* = 12 sites	Total bacteria	5.8 ± 0.9	5.1 ± 0.8	0.0050^†^
	*P. gingivalis *	4.3 ± 1.9	3.4 ± 1.7	0.0076^†^
	*P. intermedia *	3.1 ± 1.6	3.0 ± 1.7	0.5933
	*T. forsythia *	4.2 ± 1.1	3.2 ± 0.9	0.0076^†^

Values are expressed as mean ± SD (log⁡10/mL). EO: essential oil-containing antiseptic; *P. gingivalis*: *Porphyromonas gingivalis*; *P. intermedia*: * Prevotella intermedia*; *T. forsythia*: *Tannerella forsythia*. Wilcoxon signed-rank test was used for statistical analysis (*P* < 0.05, *P < 0.01, ^†^decrease).

**Table 4:** Comparison of subgingival bacterial counts in deep pockets (PPD ≥ 6 mm).

		Baseline	After 7 days	*P* value
Control group *n* = 22 sites	Total bacteria	5.8 ± 1.0	6.1 ± 1.0	0.0151^‡^
	*P. gingivalis *	4.5 ± 1.3	4.9 ± 1.3	0.0104^‡^
	*P. intermedia *	2.9 ± 1.6	3.2 ± 1.9	0.1843
	*T. forsythia *	4.2 ± 1.2	4.5 ± 1.4	0.1069

Saline group *n* = 18 sites	Total bacteria	5.9 ± 0.9	5.8 ± 0.9	0.5692
	*P. gingivalis *	4.1 ± 1.4	3.5 ± 1.6	0.0663
	*P. intermedia *	2.7 ± 1.4	3.0 ± 1.4	0.2550
	*T. forsythia *	4.1 ± 0.9	3.8 ± 1.0	0.2006

EO group *n* = 18 sites	Total bacteria	6.0 ± 0.7	5.2 ± 1.0	0.0165*
	*P. gingivalis *	4.6 ± 1.5	3.8 ± 2.0	0.0038^†^
	*P. intermedia *	2.3 ± 1.8	2.0 ± 1.7	0.3063
	*T. forsythia *	4.8 ± 0.9	3.8 ± 1.5	0.0191*

Values are expressed as mean ± SD (log⁡10/mL). EO: essential oil-containing antiseptic; *P. gingivalis*: *Porphyromonas gingivalis*; *P. intermedia*: * Prevotella intermedia*; *T. forsythia*: *Tannerella forsythia*. Wilcoxon signed-rank test was used for statistical analysis (*P* < 0.05, *P < 0.01, ^†^decrease. *P* < 0.05, ^‡^increase).
